# Reference ranges of tricuspid annulus geometry in healthy adults using a dedicated three-dimensional echocardiography software package

**DOI:** 10.3389/fcvm.2022.1011931

**Published:** 2022-09-13

**Authors:** Denisa Muraru, Mara Gavazzoni, Francesca Heilbron, Diana J. Mihalcea, Andrada C. Guta, Noela Radu, Giuseppe Muscogiuri, Michele Tomaselli, Sandro Sironi, Gianfranco Parati, Luigi P. Badano

**Affiliations:** ^1^Department of Medicine and Surgery, University of Milano-Bicocca, Milan, Italy; ^2^Department of Cardiology, Istituto Auxologico Italiano, Istituto di Ricovero e Cura a Carattere Scientifico (IRCCS), Milan, Italy; ^3^University of Medicine and Pharmacy Carol Davila Bucharest, Emergency University Hospital Bucharest, Bucharest, Romania; ^4^Department of Cardiology, Methodist DeBakey Heart Center, Houston Methodist Hospital, Houston, TX, United States; ^5^Radiology Department, Azienda Socio Sanitaria Territoriale (ASST) Papa Giovanni XXIII Hospital, Bergamo, Italy

**Keywords:** three-dimensional echocardiography, two-dimensional echocardiography, cardiac computed tomography, tricuspid valve, tricuspid annulus, reference values, healthy volunteers

## Abstract

**Background:**

Tricuspid annulus (TA) sizing is essential for planning percutaneous or surgical tricuspid procedures. According to current guidelines, TA linear dimension should be assessed using two-dimensional echocardiography (2DE). However, TA is a complex three-dimensional (3D) structure.

**Aim:**

Identify the reference values for TA geometry and dynamics and its physiological determinants using a commercially available three-dimensional echocardiography (3DE) software package dedicated to the tricuspid valve (4D AutoTVQ, GE).

**Methods:**

A total of 254 healthy volunteers (113 men, 47 ± 11 years) were evaluated using 2DE and 3DE. TA 3D area, perimeter, diameters, and sphericity index were assessed at mid-systole, early- and end-diastole. Right atrial (RA) and ventricular (RV) end-diastolic and end-systolic volumes were also measured by 3DE.

**Results:**

The feasibility of the 3DE analysis of TA was 90%. TA 3D area, perimeter, and diameters were largest at end-diastole and smallest at mid-systole. Reference values of TA at end-diastole were 9.6 ± 2.1 cm^2^ for the area, 11.2 ± 1.2 cm for perimeter, and 38 ± 4 mm, 31 ± 4 mm, 33 ± 4 mm, and 34 ± 5 mm for major, minor, 4-chamber and 2-chamber diameters, respectively. TA end-diastolic sphericity index was 81 ± 11%. All TA parameters were correlated with body surface area (BSA) (*r* from 0.42 to 0.58, *p* < 0.001). TA 3D area and 4-chamber diameter were significantly larger in men than in women, independent of BSA (*p* < 0.0001). There was no significant relationship between TA metrics with age, except for the TA minor diameter (*r* = −0.17, *p* < 0.05). When measured by 2DE in 4-chamber (29 ± 5 mm) and RV-focused (30 ± 5 mm) views, both TA diameters resulted significantly smaller than the 4-chamber (33 ± 4 mm; *p* < 0.0001), and the major TA diameters (38 ± 4 mm; *p* < 0.0001) measured by 3DE. At multivariable linear regression analysis, RA maximal volume was independently associated with both TA 3D area at mid-systole (*R*^2^ = 0.511, *p* < 0.0001) and end-diastole (*R*^2^ = 0.506, *p* < 0.0001), whereas BSA (*R*^2^ = 0.526, *p* < 0.0001) was associated only to mid-systolic TA 3D area.

**Conclusions:**

Reference values for TA metrics should be sex-specific and indexed to BSA. 2DE underestimates actual 3DE TA dimensions. RA maximum volume was the only independent echocardiographic parameter associated with TA 3D area in healthy subjects.

## Introduction

The dilation of the tricuspid annulus (TA) is one of the main determinants of the development and the severity of secondary tricuspid regurgitation (STR), particularly in patients with the atrial form of STR (1–4). Moreover, the size of the TA is critical in determining the need for concomitant tricuspid valve (TV) interventions in patients undergoing left-sided valve surgery (5, 6) and in the patient selection for transcatheter procedures to repair the regurgitant TV (7).

Three-dimensional visualization of the TA can be obtained from cardiac magnetic resonance (CMR) and cardiac computed tomography (CCT), but the relatively limited access in some centers coupled with the need for dedicated imaging protocols for the right heart with lengthy acquisition and post-processing, and the use of contrast media or radiation restrict the application of these imaging modalities mainly to patients undergoing evaluation for possible TV interventions. In clinical routine, echocardiography is the most frequently used imaging technique to assess patients with heart valve diseases (8). Current guidelines for TV repair recommend TA sizing by two-dimensional transthoracic echocardiography (2DE) by measuring the TA diameter during diastole from an apical 4-chamber view (5, 6). However, TA is a highly dynamic, saddle-shaped structure and, because of this complex three-dimensional (3D) geometry, it is unlikely that a single linear dimension can account for its actual size (9–11). Moreover, when the TA dilates, it does mostly in the anteroposterior direction, a direction that is not explored in the conventional 2DE apical 4-chamber view (10). Accordingly, the most suitable echocardiographic technique to obtain accurate measurements of TA size is 3D echocardiography (3DE) (12, 13).

The main issues limiting the clinical use of 3DE to measure TA size and its dynamic changes during the cardiac cycle were the lack of a dedicated software package for the TV and the consequently limited availability of reference values for the parameters describing the TA geometry based on 3DE semi-automated measurements (9). Recently, a software package specifically designed to analyze TV on 3DE data sets has become commercially available. However, the accuracy of the TA measurements using this software package, as well as the TA reference values by 3DE, have not been reported so far.

Accordingly, we sought to: (i) use CCT to test the accuracy of the measurements of the TA geometry obtained with a commercially available 3DE software package dedicated to the TV; (ii) describe normal TA geometry and changes during the cardiac cycle in a relatively large cohort of healthy volunteers; (iii) explore the impact of age, sex, body size, and dimensions of right cardiac chambers on TA geometry; and (iv) compare TA diameters obtained with the new 3DE software package with those measured using conventional 2DE according to current recommendations.

## Methods

### Study population

#### Validation study

To test the accuracy of the 3DE measurements of TA geometry, we prospectively enrolled patients who underwent clinically indicated CCT and agreed to undergo a 3DE study immediately after the CCT. Patients with impaired renal function, contraindication to administration of contrast agent, pregnancy, cardiac arrhythmias, poor-quality echocardiographic images from the apical window, or inability to sustain breath-hold were excluded from the study.

#### Normative study

To assess the 3DE reference values for TA, we used a dedicated software package for 3DE analysis of TV (4D Auto TVQ GE Vingmed, Horten, Norway) to reanalyze the 3DE data sets of the healthy volunteers prospectively included in the “Padua 3D Echo Normal” study (9, 14–16). The study was approved by the Istituto Auxologico Ethics Committee (record #2020_04_21_06, approved on April 21, 2020). The need for patient written informed consent was waived due to the retrospective nature of the study. Inclusion criteria were age >17 years, no cardiovascular risk factors (smoking, arterial hypertension, dyslipidemia, diabetes), no prior history or symptoms of cardiovascular or lung disease, no cardioactive or vasoactive medication, normal physical examination, and normal electrocardiography. Exclusion criteria were athletic training, pregnancy, obesity, valvular heart disease (stenosis and more than mild regurgitation), pulmonary artery systolic pressure higher than 35 mmHg, first detected cardiac abnormalities at 2DE, poor apical acoustic window, or incomplete 3DE study. Blood pressure was measured at the beginning of the echocardiographic examination.

#### 2D echocardiography

Conventional 2DE was performed using either Vivid E9 or E95 scanners (GE Vingmed, Horten, Norway) equipped with an M5S probe. TA diameter was measured from both the standard apical 4-chamber and the right ventricle (RV)—focused apical view at three time points during the cardiac cycle, identified based on TV mechanics as follows: early-diastole (the first frame after TV opening), end-diastole (the last frame before TV closure), and mid-systole (the frame midway between the TV closure time and end-systole, where end-systole was defined as the last frame before TV opening).

#### 3D echocardiography

**Figure 1:**
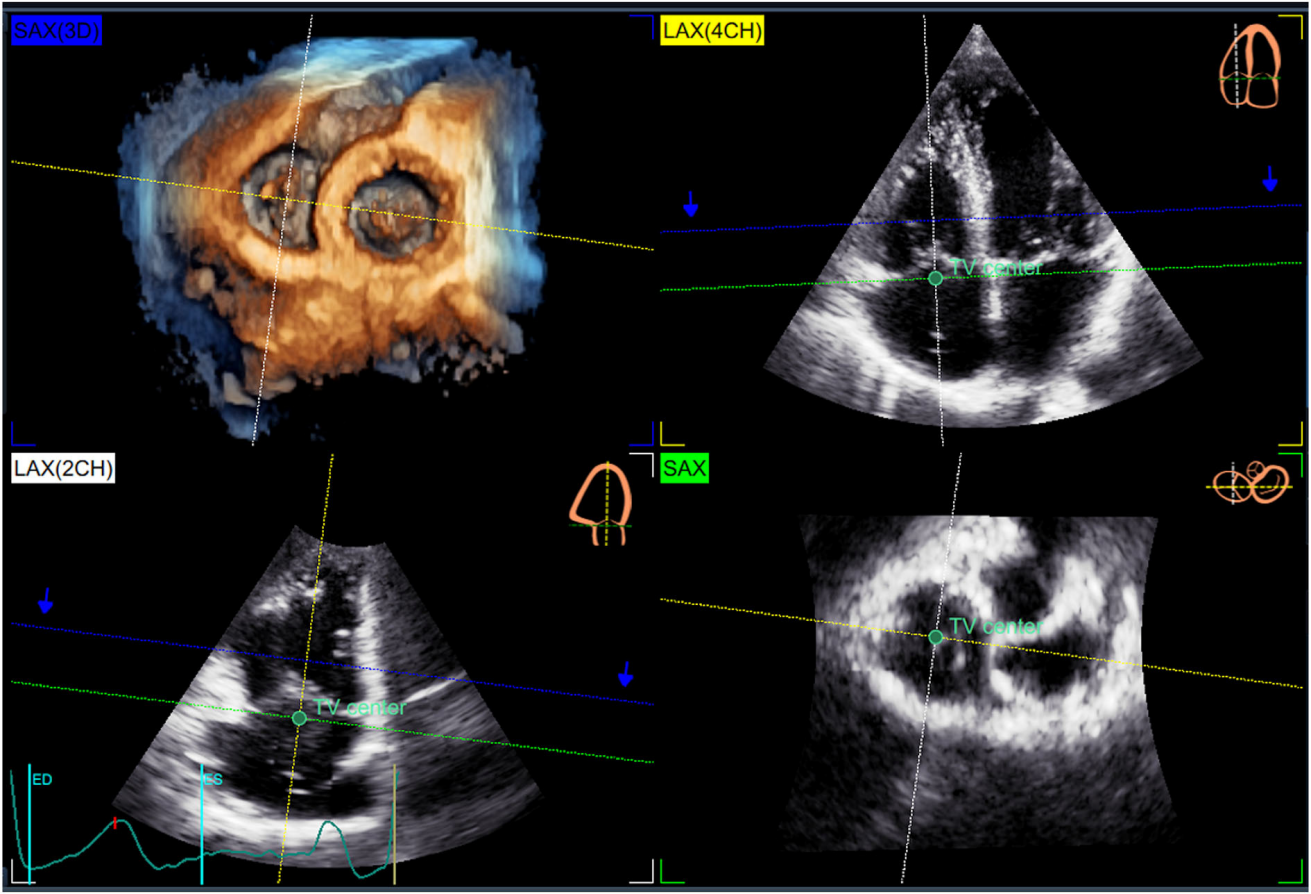
Automatic slicing of the three-dimensional echocardiography dataset to start the quantitative analysis of the tricuspid annulus. The 3DE data set [SAX(3D) panel] is automatically sliced to obtain 3 cut planes corresponding to the apical 4-chamber view [LAX(4CH) panel], its orthogonal longitudinal cut-plan [LAX(2CH) panel], and a transversal cut plane (SAX Panel). The transverse cut plane is positioned at the hinge points of the tricuspid valve leaflets with the annulus (green broken dashed line), and the longitudinal cut planes (yellow and white broken dashed lines) positioned to intersect the center of the TA (green dot) and the right ventricular apex.

**Figure 2:**
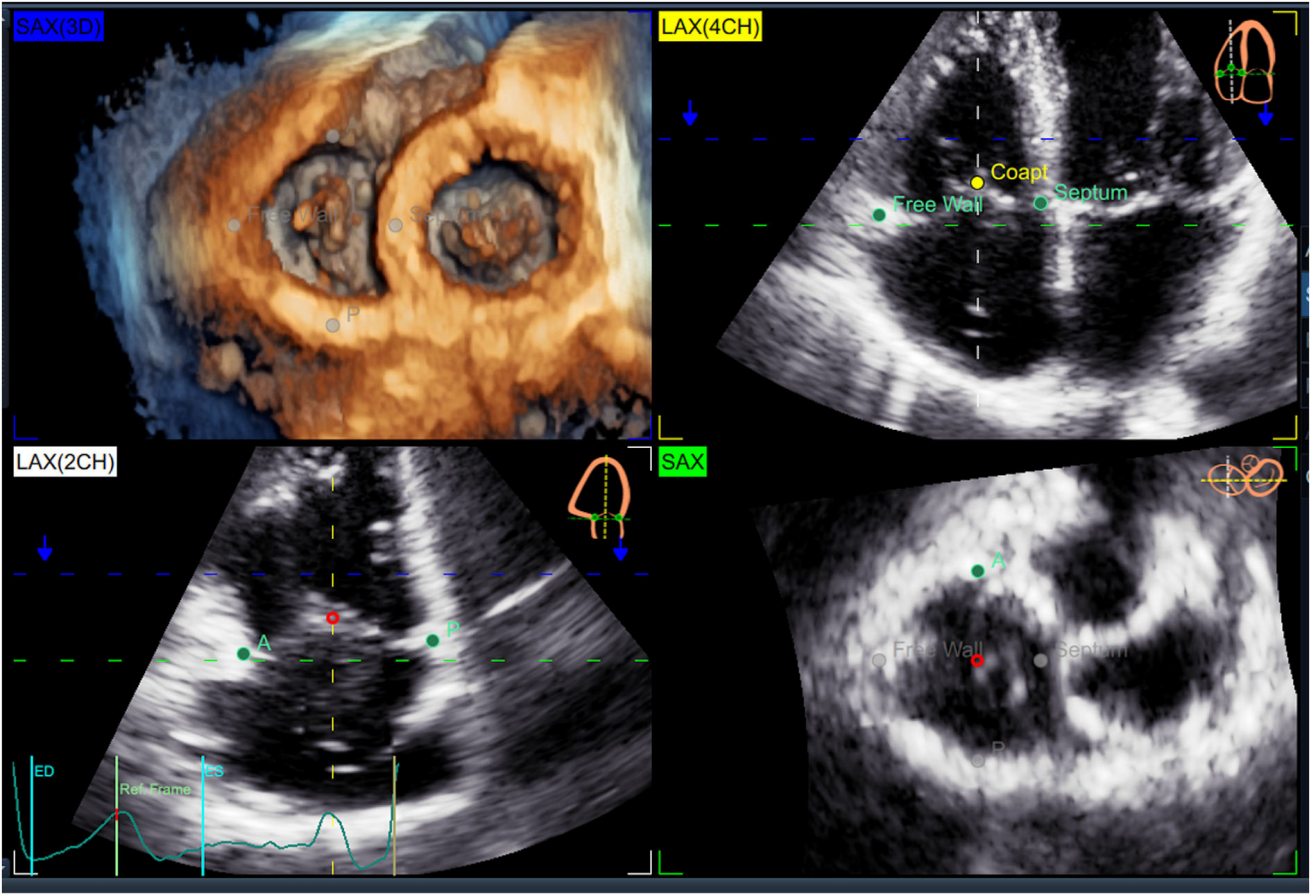
Initialization of the 4D Auto4DTVQ software package. Initialization consists of the manual identification of the tricuspid valve leaflet hinge points [RV free wall and septum in the 4-chamber view LAX(4CH)], and the anterior (A) and the posterior (P) in the orthogonal longitudinal view [LAX(2CH)], and the TV leaflet coaptation point (Coapt) in the reference planes.

**Figure 3:**
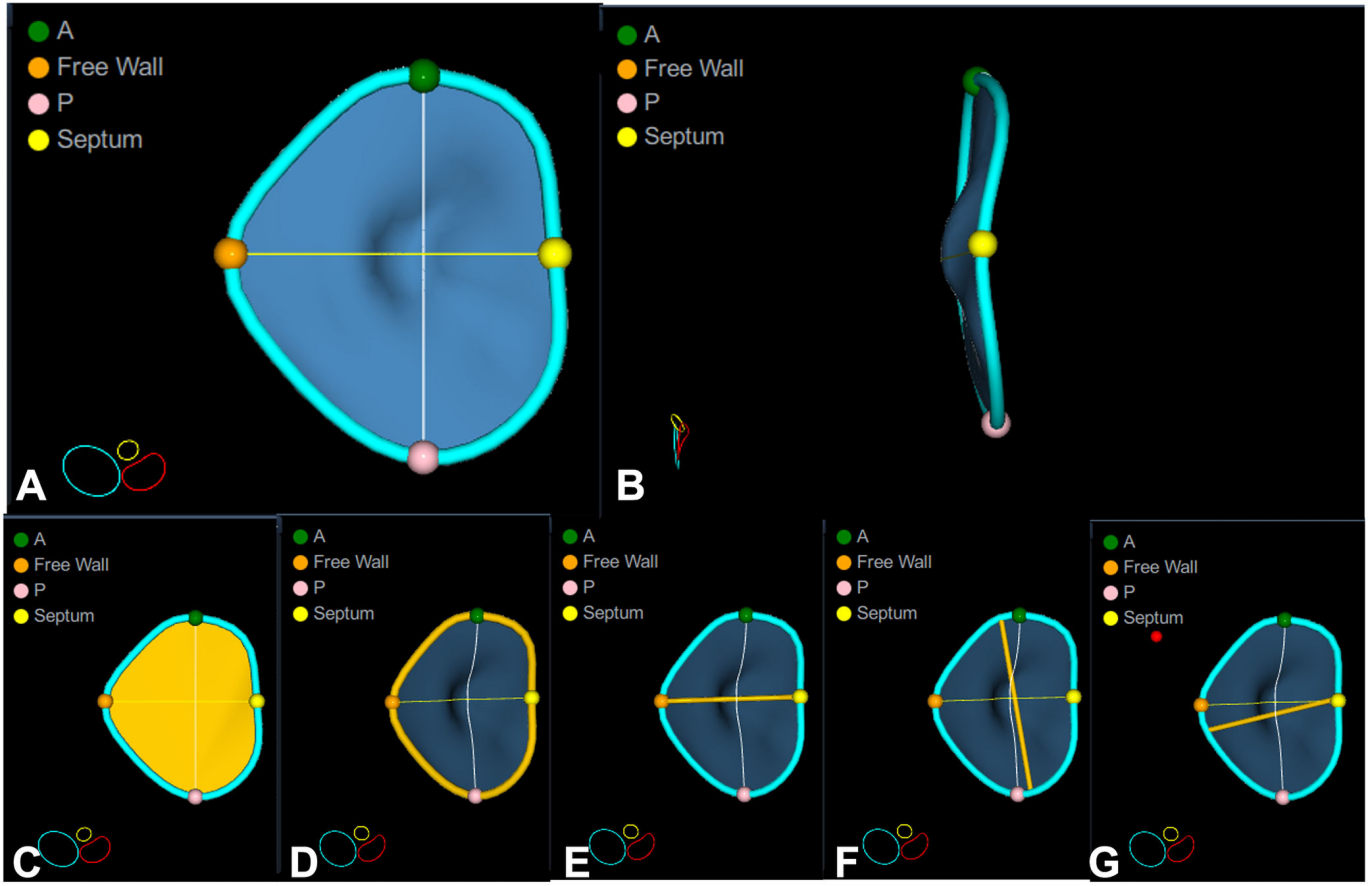
Quantitative analysis of the tricuspid annulus geometry. Surface rendering of a normal tricuspid valve that allows appreciating its complex three-dimensional shape: D-shaped when seen en-face from the ventricular perspective **(A)**, and saddle-shaped with higher points in anteroseptal and posterolateral portions and lower points anterolateral and posteroseptal portions when seen from the lateral point of view **(B)**. The most useful quantitative parameters are shown in the bottom panels: annulus area **(C)**, annulus perimeter **(D)**, 4-chamber diameter **(E)**, major **(F)**, and minor **(G)** diameters/axes. Note that the 4-chamber diameter (3.6 cm) is significantly smaller than the actual major axis (4.3 cm), and it does not account for either the major or the minor anatomical diameter of the tricuspid annulus. A, anterior point; Free wall, right ventricular free wall point; P, posterior point; Septum, interventricular septal point.

Datasets were acquired using either the 4V-D or the 4Vc-D matrix array transducer. Separate full-volume 3DE data sets of the right atrium (RA), RV, and TV were obtained from the RV-focused apical view by combining four to six electrocardiographically gated consecutive subvolumes during breath-hold (17). The temporal resolution of the 3DE data sets was optimized by reducing the sector depth and the volume size, achieving a mean volume rate of 32 ± 5 vps. All 2DE and 3DE data sets were stored digitally and exported for offline analysis. RV end-diastolic and end-systolic volumes and ejection fraction (RVEF) were measured using a commercially available 3DE software package (4D RV Function 2.0 TomTec, Unterschleissheim, Germany) previously validated against CMR (18). RA maximal volume was measured with a commercially available 3DE software package designed for atrium quantification (4D Auto LAQ, GE Vingmed, Horten, Norway) previously described and validated against CMR (19). The methodology used for the 3DE acquisition of the TV and the analysis workflow of 4D AutoTVQ software have been detailed elsewhere (https://gevividultraedition.com/storage/app/media/whitepapers/4D-Auto-TVQ-whitepaper-JB03442XX.pdf). In brief, TA analysis started by identifying two-time points during the index cardiac cycle: the end-diastole (the first frame before TV closure) and the end-systole (the frame before TV opening), then the mid-systolic frame was automatically identified. The 3DE data set was automatically sliced to obtain 3 cut planes corresponding to the apical 4-chamber view, its orthogonal longitudinal cut-plan, and a transversal cut plane (Figure 1). Both the position and the spatial orientation of the cut planes were manually adjusted to obtain the view of interest in both 2D and 3D images. In particular, the transverse cut plane was positioned at the hinge points of the TV leaflets with the TA, and the longitudinal cut planes were positioned to intersect the center of the TA and the RV apex (Figure 1). Initialization of the TA was manually performed by identifying the TV leaflet hinge points (RV free wall and septum in the 4-chamber view, and the anterior and the posterior walls in the orthogonal longitudinal view), and the TV leaflet coaptation point in the reference planes (Figure 2). Then, the software package automatically created a surface rendering of the TA and tracked it throughout the cardiac cycle, also giving the operator the possibility to edit it manually if needed. The process was repeated at three-time points during the cardiac cycle: mid-systole, early-diastole, and end-diastole, identified as described above. The following quantitative parameters were obtained from the 3DE meshes of the TV: TA area; TA perimeter; 4-chamber TA diameter (the distance between the septum and RV free wall); 2-chamber TA diameter (the distance between RV anterior and posterior wall); major TA diameter (the largest diameter of the TA); minor TA diameter (the smallest diameter of the TA); sphericity index (the ratio between the minor and the major diameters); TA excursion (absolute displacement of the cardiac annulus throughout the cardiac cycle), TA coaptation height (distance between the coaptation point and 4-chamber TA annulus diameter); and TA tenting volume (volume encompassed by the TV leaflets and the TA surface in mid-systole) (Figure 3).

### Reproducibility analysis

Intra- and inter-observer reproducibility of TA parameters measured by 3DE were tested by computing intraclass correlations and coefficients of variation. Intra-observer variability was tested by re-analyzing the same beat on 30 3DE data sets of the TV 1 week apart by the same researcher (M.D.) blinded from the initial measurements. The inter-observer variability was tested by having the same datasets analyzed by a different researcher (G.A.C.) who was not aware of the results of the other.

### Cardiac computed tomography

**Figure 4:**
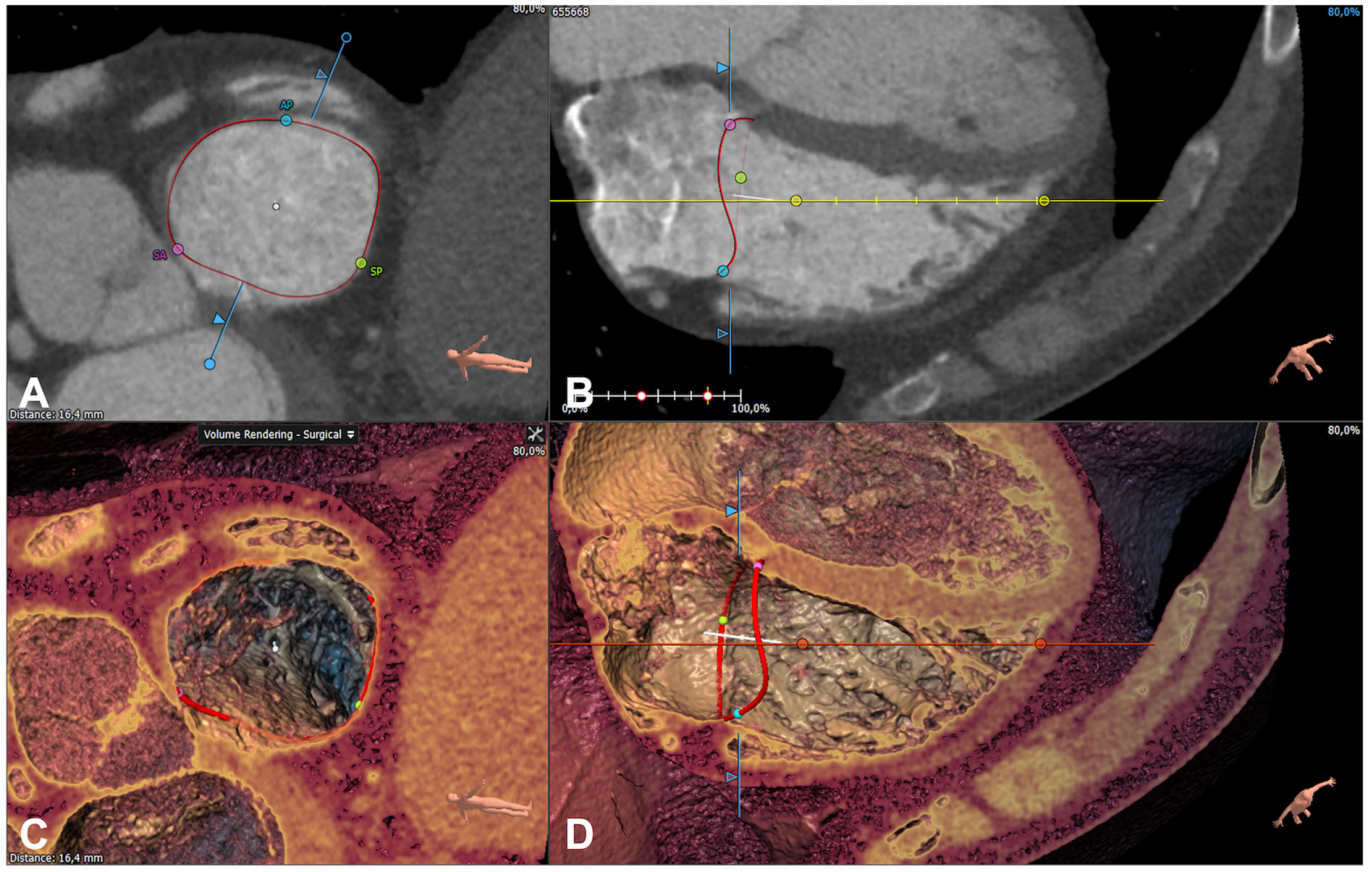
Quantitative analysis of the tricuspid annulus by cardiac computed tomography. The reconstruction of the tricuspid annulus (red circle) using 3 mensio (Pie Medical Imaging, Maastricht, The Netherlands) shown on both multiplanar [short axis **(A)** and 4-chamber **(B)**] and volume rendered [short axis **(C)** and 4-chamber **(D)**] reconstructions.

Cardiac CT angiographies were acquired using a CT Revolution scanner (GE Healthcare, Milwaukee, Wisconsin) and a dedicated protocol focusing on right chamber opacification (20). CCT was acquired using retrospective gating (0–100% of R-R interval) using the following parameters: 256 × 0.625 mm; voxel size, 0.625 mm, spatial resolution along the X-Y planes, 0.23 mm; gantry rotation time, 280 ms. Tube current and tube voltage were adapted based on the patient's body mass index, as previously described (21). Injection protocol was done by the injection in the antecubital vein of 60 ml of iodine contrast agent (Iomeron, Bracco, Milan, Italy) at a flow rate of 5 ml/s followed by a 20 ml mixture of 50:50% of contrast and saline, followed by 20 ml of saline (22). CCT was acquired after a threshold of 150 HU was reached in the left ventricle (22). Subsequently, the images were reconstructed at each 10% of R-R interval. The reconstructions were transferred to an external workstation equipped with dedicated software for the 3D reconstruction and quantitative analysis of TA (3mensio, Pie Medical Imaging, Maastricht, The Netherlands). Firstly, the end-diastolic and end-systolic phases were identified. Subsequently, multiplanar reconstructions of TA were obtained, and the dimensions of TA were calculated based on the 3D model of TA superimposed on the CCT images (Figure 4).

### Statistical analysis

All data were analyzed using SPSS version 28.0 (IBM, Chicago, Illinois). Continuous variables were expressed as mean ± SD, and categorical variables were summarized as numbers or percentages, as appropriate. The normality of continuous data was verified using the Shapiro-Wilk test. Intergroup comparison of baseline and echocardiographic characteristics was made using independent Student's *t*-tests or analysis of variance (ANOVA), as appropriate. Pairwise comparisons of means after a significant ANOVA were made by *post-hoc* Tukey honest significant difference test. Bland-Altman plots were developed to assess the agreement between the TA area, perimeter, and major axis length measured using 3DE and CCT. Correlations between normally distributed variables were tested using Pearson analysis. Multivariate linear regression analyses were performed to identify the correlates of the TA area. A *p*-value < 0.05 was considered significant.

## Results

### Validation study

**Figure 5:**
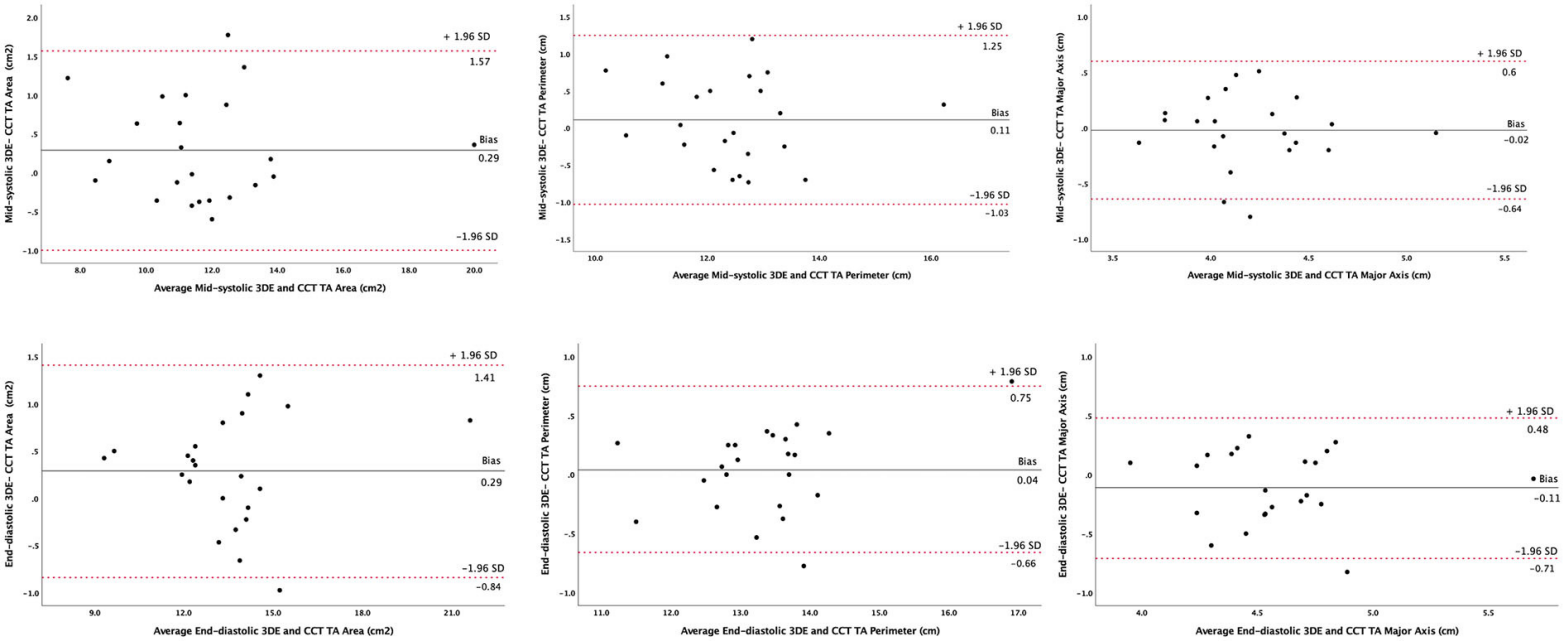
Agreement between three-dimensional echocardiography and cardiac computed tomography in measuring tricuspid annulus geometry parameters. Bland-Altman plots analyzing tricuspid annulus area (left panels), perimeter (central panels), and major axis (right panels) at mid-systole (upper panels) and end-diastole (lower panels). 3DE, three-dimensional echocardiography; CCT, cardiac computed tomography; SD, standard deviation; TA, tricuspid annulus.

Thirty patients (11 men, 61 ± 10 years) were enrolled in the validation study of the 3DE parameters of TA. Three patients with artifacts on CCT images and two patients with the insufficient quality of the 3DE data sets were excluded from the comparison. Although TA parameters were statistically larger (*p* < 000.1) when measured by 3DE compared to CCT both at mid-systole (TA 3D area 11.9 ± 2.4 vs. 11.6 ± 2.4 cm^2^, TA perimeter 12.5 ± 1.2 vs. 12.4 ± 1.3 cm, and TA major axis 4.2 ± 0.4 vs. 4.2 ± 0.4 cm for 3DE vs. CCT, respectively) and at end-diastole (TA 3D area 13.7 ± 2.3 vs. 13.4 ± 1.1 cm^2^, TA perimeter 13.4 ± 1.1 vs. 13.3 ± 1.0 cm, and TA major axis 4.5 ± 0.4 vs. 4.6 ± 0.4 cm for 3DE vs. CCT, respectively), the differences between the two imaging modalities were not clinically relevant. Small biases and reasonable limits of agreement were found between 3DE and CCT measurements of the TA area, perimeter, and major axis at mid-systole and end-diastole (Figure 5).

### Normative study

A total of 254 healthy volunteers (113 men, mean age 47 ± 11 years) were first selected from the “Padua 3D Echo Normal” database. Among these, TA analysis was feasible in 228 subjects (99 men, mean age 45 ± 16 years) representing the final normative study group (feasibility of 4D AutoTVQ = 90%). Their demographics, clinical characteristics, and right heart parameters are summarized in Table 1. Age and heart rate were similar between the sexes. Women had significantly smaller body surface area (BSA) and lower blood pressure values than men (*p* < 0.001). Indexed RA volumes, RV end-diastolic, and end-systolic volumes were larger in men, whereas RVEF was higher in women (*p* < 0.002; Table 1).

**Table 1:** Demographics, clinical characteristics, and right heart parameters of the study group.

**Parameters**	**All (*n* = 228)**	**Men (*n* = 99)**	**Women (*n* = 129)**
Age (years)	45 ± 16	44 ± 17	45 ± 15
Height (cm)	170 ± 10	173 ± 9	167 ± 9*
Weight (kg)	66 ± 11	69 ± 10	63 ± 11*
Body surface area (m^2^)	1.7 ± 0.2	1.8 ± 0.2	1.7 ± 0.2*
Heart rate (beats/min)	68 ± 11	69 ± 11	68 ± 11
Systolic blood pressure (mmHg)	120 ± 13	124 ± 12	117 ± 14*
Diastolic blood pressure (mmHg)	72 ± 9	74 ± 8	71 ± 9*
Maximum right atrial volume (ml/m^2^)	29 ± 8	32 ± 8	28 ± 7*
Minimum right atrial volume (ml/m^2^)	11 ± 4	12 ± 4	10 ± 3*
Right ventricular end-diastolic volume (ml/m^2^)	57 ± 15	65 ± 13	52 ± 13*
Right ventricular end-systolic volume (ml/m^2^)	23 ± 8	27 ± 7	20 ± 6*
Right ventricular ejection fraction (%)	59 ± 5	57 ± 5	60 ± 5*

Values are shown as mean ± SD or percentage.

^*^p < 0.005 men vs. women; Right ventricular and atrial volumes were measured by three-dimensional echocardiography.

The TA post-processing using 4D AutoTVQ at mid-systole, early-diastole, and end-diastole took 4–5 min on average. All TA geometry parameters showed significant correlations with body size measurements (BSA, *r* = 0.42–0.58; height, *r* = 0.37–0.52) (all *p* < 0.0001). Conversely, there was no significant correlation with age (except for the minor diameter, *r* = −0.17, *p* < 0.05). Moreover, when the comparison of the various parameters of TA geometry was made among the five selected age groups (<30, 30–40, 40–50, 50–60, and >60 years), there were no significant differences at any time point during the cardiac cycle.

### 3D TA geometry changes during cardiac cycle

**Figure 6:**
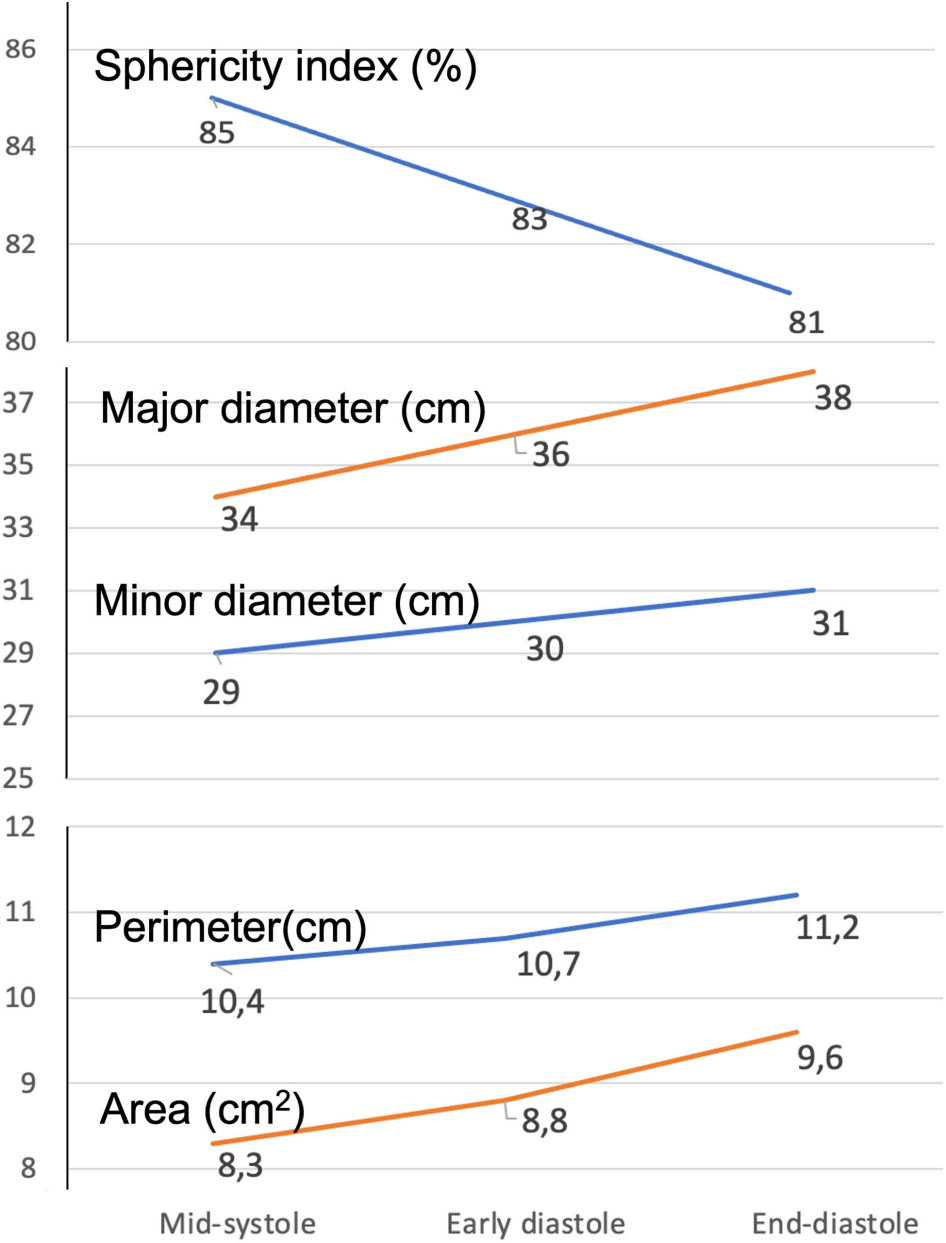
Dynamics of the tricuspid annulus during the cardiac cycle. Change of the various parameters describing tricuspid annulus geometry from mid-systole to end-diastole.

All parameters describing 3DE TA geometry changed significantly during the cardiac cycle in both sexes (all *p* < 0.005; Table 2). TA area, perimeter, and diameters (4-chamber, 2-chamber, major, and minor) were at their minimum at mid-systole, then increased during early-diastole, reaching their maximum value at end-diastole (Figure 6). TA sphericity index decreased progressively throughout the cardiac cycle, with TA having the most oval shape at end-diastole (Figure 6).

**Table 2:** Tricuspid annulus parameters measured by three-dimensional echocardiography.

	**Mid-systole**				**Early diastole**				**End-diastole**			
	**Men**	**ULN**	**Women**	**ULN**	**Men**	**ULN**	**Women**	**ULN**	**Men**	**ULN**	**Women**	**ULN**
**Absolute values**												
Area (cm^2^)	9.2 ± 2.2	13.1	7.7 ± 1.7*	10.2	9.7 ± 0.2	13.5	8.1 ± 1.7*	11.2	10.4 ± 2.2	13.8	9.1 ± 1.7*	11.7
Perimeter (cm)	10.9 ± 1.3	13.4	10 ± 1.1*	11.5	11.4 ± 2.0	13.4	10.3 ± 1.6*	12	11.6 ± 1.2	13.7	10.9 ± 1.2*	12.5
4-chamber diameter (mm)	33 ± 5	41	29 ± 4*	36	35 ± 4	41	29 ± 4*	37	36 ± 4	42	32 ± 3*	37
2-chamber diameter (mm)	31 ± 5	41	31 ± 5	38	32 ± 5	40	32 ± 5	40	35 ± 5	43	34 ± 5	42
Major diameter (mm)	36 ± 4	43	33 ± 4*	40	38 ± 4	44	35 ± 4*	42	39 ± 4	46	37 ± 4*	43
Minor diameter (mm)	30 ± 5	40	27 ± 4*	34	31 ± 5	39	28 ± 4*	35	33 ± 5	41	30 ± 3*	35
Sphericity index (%)	84 ± 11	98	83 ± 10	99	82 ± 11	96	80 ± 12	96	84 ± 10	99	82 ± 9	96
Excursion (mm)	15 ± 3	19	14 ± 3	19								
Coaptation height (mm)	8 ± 2	12	7 ± 2*	11								
Maximum tenting height (mm)	7 ± 2	9	6 ± 2*	9								
Tenting volume (ml)	2 ± 1	3	1.2 ± 0.5*	2								
**Values indexed to body surface area**												
Area (cm^2^)	5.1 ± 1.2	7.5	4.5 ± 1*	6.0	5.3 ± 1.2	7.3	4.8 ± 1*	6.3	5.7 ± 1.2	8.4	5.3 ± 1*	6.9
Perimeter (cm)	6 ± 0.8	7.7	5.9 ± 0.8	7.2	6.3 ± 1.1	7.7	6.1 ± 1.1	7.3	6.4 ± 0.8	8.0	6.4 ± 0.9	7.8
4-chamber diameter (mm)	18 ± 3	23	17 ± 3*	22	19 ± 3	24	17 ± 3*	21	20 ± 3	25	19 ± 3*	23
2-chamber diameter (mm)	17 ± 3	24	18 ± 3	22	18 ± 3	23	19 ± 3	24	19 ± 3	24	20 ± 3	25
Major diameter (mm)	20 ± 3	25	19 ± 3	23	21 ± 3	25	21 ± 3	25	21 ± 3	27	21 ± 3	26
Minor diameter (mm)	17 ± 3	22	16 ± 3	21	17 ± 3	22	16 ± 3*	21	18 ± 3	23	18 ± 3	22

^*^p < 0.001 men vs. women.

ULN, Upper limit of normality (i.e., upper bound of the 95% confidence interval of the mean).

### Sex-related differences in 3D TA parameters

Men had larger absolute TA area, perimeter, and diameters than women, irrespective of the measurement timing (*p* < 0.0001 for all comparisons of men vs. women at mid-systolic, early- and end-diastole). Conversely, TA excursion and sphericity index was similar between the sexes (Table 2). After indexing to BSA, the sex differences persisted for TA area and 4-chamber diameter only. Moreover, men had higher coaptation height, maximum tenting height, and tenting volumes than women (*p* < 0.005; Table 2).

### 3D vs. 2D TA parameters

Both at mid-systole and at end-diastole, TA diameters obtained from the apical RV-focused view were significantly larger than those obtained from the apical 4-chamber view (2.8 ± 0.4 cm vs. 2.7 ± 0.5 cm, and 3.0 ± 0.5 cm vs. 2.8 ± 0.5 cm; respectively, all *p* < 0.0001). Moreover, men had larger 2DE TA diameters than women, irrespective of the 2DE view used for TA sizing (Table 3). Only the diameters obtained from the 2DE RV-focused view during the cardiac cycle showed the expected increase from mid-systole to end-diastole.

**Table 3:** Tricuspid annulus geometry parameters measured using two-dimensional echocardiography.

**2DE parameters**	**Mid-systole**				**End-diastole**			
	**Men**	**ULN**	**Women**	**ULN**	**Men**	**ULN**	**Women**	**ULN**
**Absolute values**								
Apical 4-chamber diameter (mm)	31 ± 5	40	25 ± 4*	37	31 ± 5*	37	27 ± 5*	34
Apical RV-focused diameter (mm)	31 ± 4	38	26 ± 3*	31	32 ± 5*	41	28 ± 45*	38
**Value indexed to body surface area**												
Apical 4-chamber diameter (mm/m^2^)	17 ± 3	23	16 ± 3	22	17 ± 3	21	16 ± 3*	22
Apical RV-focused diameter (mm/m^2^)	17 ± 3	21	16 ± 2*	19	18 ± 4	24	17 ± 3	22

^*^p < 0.001 men vs. women.

ULN, Upper limit of normality (i.e., upper bound of the 95% confidence interval of the mean).

The 2DE TA diameters measured in apical 4-chamber and RV-focused views were significantly smaller than the corresponding 4-chamber diameter obtained from the 3DE dataset both at end-diastole (2.9 ± 0.5 cm, 3.0 ± 0.3 cm, and 3.4 ± 0.4 cm, respectively, *p* < 0.0001) and mid-systole (2.8 ± 0.5 cm, 2.9 ± 0.5 cm, and 3.0 ± 0.5 cm, respectively, *p* < 0.001). 3D TA areas measured at early diastolic, end-diastolic and mid-systolic frames showed only a modest correlation with the TA diameters obtained from the 2DE apical 4-chamber (*r* = 0.45; *r* = 0.41; and *r* = 0.39, respectively) and RV-focused view (*r* = 0.51; *r* = 0.48; and *r* = 0.48, respectively).

Seventeen subjects (6%) had an end-diastolic TA diameter obtained from the 2DE apical 4-chamber view larger than 21 mm/m^2^.

### Correlations between the 2DE and the 3DE TA parameters and the right heart chamber size

**Figure 7:**
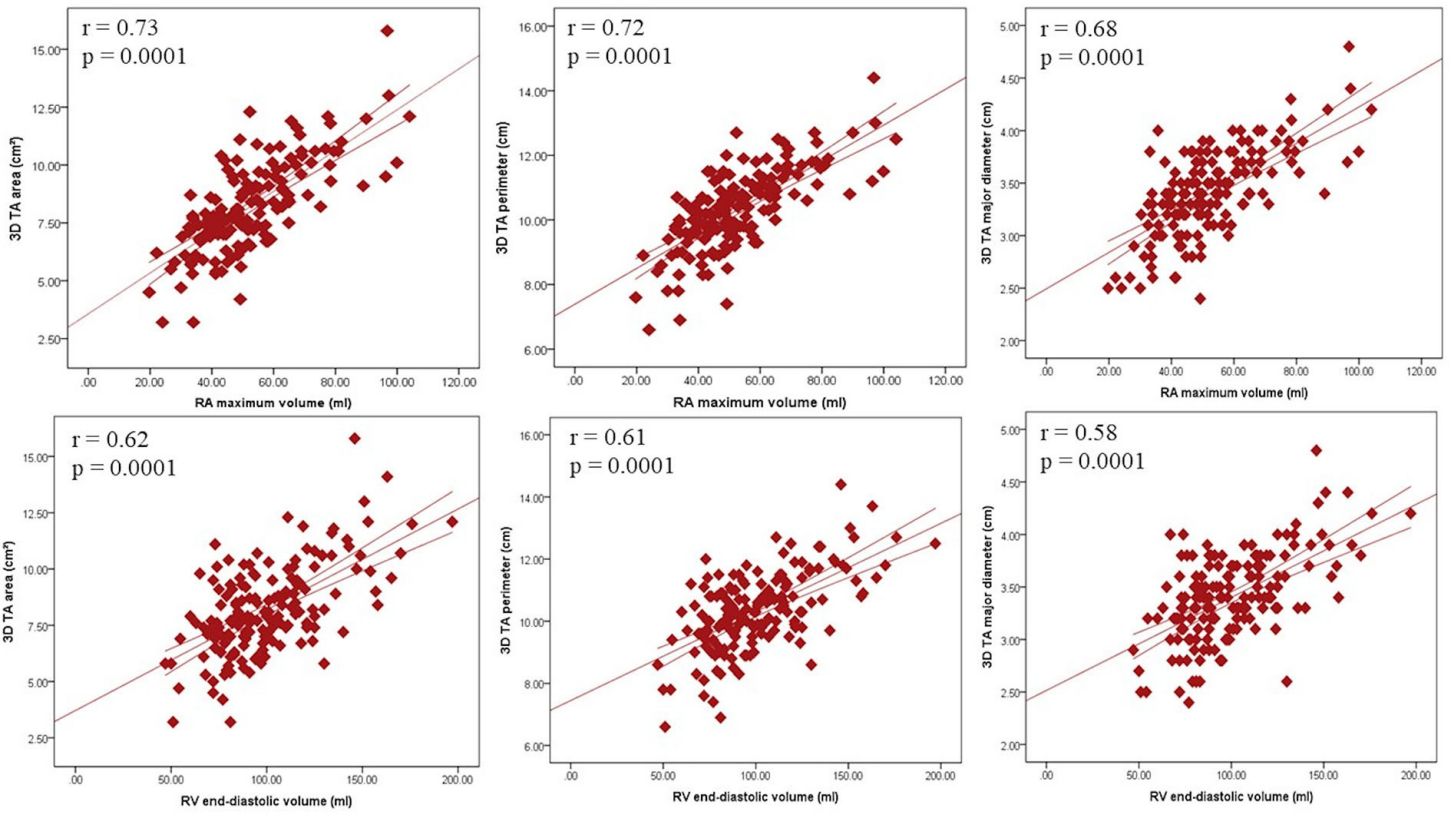
Relationship between tricuspid annulus size and the size of the right heart chambers. Correlations between tricuspid annulus area (left panels), perimeter (central panels), and major axis (right panels) with right atrial (upper panels) and ventricular (lower panels) volumes. 3D, three-dimensional; RA, right atrial; RV, right ventricular; TA, tricuspid annulus.

TA diameters measured on apical RV-focused view and 4-chamber view by 2DE had a modest correlation with RA maximal volume (*r* = 0.56 and *r* = 0.43, respectively), RV end-diastolic volume (*r* = 0.42 and *r* = 0.35, respectively) and RV end-systolic volume (*r* = 0.34 and *r* = 0.27, respectively) (all *p* < 0.0001). TA 3D area, perimeter and diameters (major, minor, and 4-chamber) were more closely correlated with RA maximal volume (*r* = 0.73; *r* = 0.72; *r* = 0.68; *r* = 0.62; and 0.59, respectively), than with RV end-diastolic volume (*r* = 0.62; *r* = 0.61; *r* = 0.58; *r* = 0.49; and *r* = 0.48, respectively) and RV end-systolic volume (*r* = 0.57; *r* = 0.56; *r* = 0.55; *r* = 42; and *r* = 0.50, respectively) (*p* < 0.0001; Figure 7). By multivariable linear regression analysis, in a model that included RA maximal and minimal volumes, RV end-diastolic and end-systolic volumes, sex, age, and BSA, RA maximal volume was independently associated with TA 3D area both at mid-systole (*R*^2^ = 0.511, *p* < 0.0001) and end-diastole (*R*^2^ = 0.506, *p* < 0.0001), whereas BSA (*R*^2^ = 0.526, *p* < 0.0001) was associated only to mid-systolic TA 3D area.

### Reproducibility and repeatability

Intraobserver and interobserver variability for 3DE TA parameters are presented in Table 4. TA 3D area, perimeter, diameters, sphericity index, and excursion showed good reproducibility and repeatability, whereas coaptation height, maximum tenting height, and tenting volume had less satisfactory reproducibility.

**Table 4:** Intraobserver and interobserver reproducibility of three-dimensional tricuspid annulus parameters (*n* = 20).

**Parameter**	**Intraobserver variability**	**Interobserver variability**
Annular area	±2.15%	±3.29%
Perimeter	±1.84%	±2.53%
4-chamber diameter	±3.3%	±4.75%
2-chamber diameter	±4.74%	±4.39%
Major diameter	±2.98%	±3.03%
Minor diameter	±5.86%	±6.72%
Sphericity index	±5.36%	±6.60%
Excursion	±9.39%	±9.95%
Coaptation point height	±12.62%	±14.49%
Maximum tenting height	±12.36%	±12.6%
Tenting volume	±9.19%	±14.67%

## Discussion

This is the first study specifically aimed to assess the TA geometry and its dynamic changes during the cardiac cycle in a relatively large cohort of healthy volunteers using a commercially available 3DE software package dedicated to TV analysis. The main results of our study can be summarized as follows: (i) TA area, perimeter, and major axis measured by 3DE and CCT showed good agreement with negligible bias and reasonable limits of agreement; (ii) the reference values of 3DE TA geometry metrics should be sex-specific and indexed to BSA; (iii) men had a larger TA 3D area and 4-chamber diameter than women, even after their indexation by BSA; (iv) parameters of TA size changed significantly during the cardiac cycle, reaching their minimum at mid-systole, then increasing through early-diastole and reaching their maximum values at end-diastole; (v) 2DE underestimated TA dimensions compared to 3DE; and (vi) RA maximal volume was the only independent parameter associated with 3D TA area both at end-diastole and mid-systole.

### Definition of normal TA size by cardiovascular imaging

Currently, TA dilatation is defined as a septal-lateral linear dimension >40 mm (>21 mm/m^2^ of BSA) measured using 2DE on an apical 4-chamber view during diastole (8, 23). Several echocardiographic (24, 25) and CMR (26, 27) studies have reported the normal values of the septal-lateral linear dimension of TA obtained from the 4-chamber view. However, the representativeness of the septal-lateral linear dimension in the 4-chamber apical view assumes that the TA is a circular and flat anatomical structure and that the 4-chamber view crosses this circle at its diameter. However, both anatomical and 3DE studies have demonstrated that the TA is a complex asymmetric (oval- or D-shaped) 3D structure (9, 28, 29). Moreover, there is no 2DE anatomical landmark that may allow the echocardiographer to orient the apical 4-chamber view in a consistent and reproducible way (30, 31). Finally, the septal-lateral linear dimension measured in a 4-chamber apical view underestimates the actual TA size (9, 32), and, since in patients with secondary tricuspid regurgitation, the TA dilation occurs mainly in the anteroposterior direction, the septal-lateral distance is unlikely to properly reflect the extent of actual TA dilation in these patients (33). Furthermore, there are no guideline recommendations about the timing of the diastole (early, mid or end-diastolic frames) for TA assessment, although the TA area changes significantly throughout systole and diastole (9, 11, 24, 33).

In addition, our results show that the 2DE TA diameter length also depends on the view used to obtain it. In our study subjects, the TA diameters obtained from the RV-focused apical view were systematically larger than those obtained from the conventional 4-chamber view confirming previous reports (24, 30).

Accordingly, to properly assess TA geometry, a 3D technique such as 3DE, CMR, and CCT is pivotal. The costs of the tests, the need for a large amount of acquisition time by CMR, and the radiation issues related to CCT, or post-processing time, limit CMR and CCT to patients with insufficient acoustic windows and those who are evaluated for possible TV interventions (34). In clinical routine, echocardiography is the most frequently used imaging technique to assess patients with heart valve diseases (8). Due to its position in the mediastinum, the TV is easily visualized by transthoracic echocardiography, and 3DE can provide anatomically sound images of the TV apparatus and analyze both TA geometry and dynamics without assumptions about shape and orientation (4, 12, 13, 23, 34–38). The main limitation of using 3DE to assess the TA was the lack of a dedicated software package to post-process the 3DE datasets.

### Normal TA size and shape

Quantifying TA by 3DE has been previously reported in relatively small cohorts of healthy volunteers (9, 24, 39) or “controls” used for comparison in various studies designed to assess pathological TR (29, 40, 41). To the best of our knowledge, this is the largest cohort of healthy volunteers from which the reference values of the parameters describing the geometry of the TA (identified as the hinge line of the TV leaflet attachment) have been derived using 3DE. In addition, this is the first study to use a software package developed specifically for the TV to obtain the reference values for the TA geometry. 3D TA area, diameters, and perimeter obtained from our healthy population were similar to those reported by other 3DE studies (9, 29, 39). Miglioranza et al. (24) found larger 4-chamber and 2-chamber TA diameters by 3DE than in our study, using tomographic planes of TA obtained by multiplanar reconstruction, but no indexation for BSA was reported. In our study, the upper limit of normality for TA 4-chamber diameter obtained by 3DE was 42 mm (25 mm/m^2^) for men and 27 mm (23 mm/m^2^) for women. However, these reference limits were smaller than the upper normal limit for the maximal linear dimension of TA irrespective of its orientation by 3DE: 46 mm (27 mm/m^2^) for men and 43 mm (26 mm/m^2^) for women.

In our healthy volunteers, the indexed values for the TA diameters obtained by 2DE at end-diastole were very similar to the 21 mm/m^2^ currently recommended to define TA dilation by 2DE: 37 mm (21 mm/m^2^) for men and 34 mm (22 mm/m^2^) for women. However, these limits of normality for 2DE were significantly smaller than those measured in the same time-point of the cardiac cycle by 3DE. Our findings are consistent with previous reports (9, 39–41), which demonstrated both in normal subjects and patients with TR that the 2DE diameters obtained from the conventional apical 4-chamber view underestimate the TA diameters compared with 3DE or CMR. When TA diameters were measured on the dedicated apical RV-focused view by 2DE [41 mm (24 mm/m^2^) for men and 38 mm (22 mm/m^2^) for women], the upper limits of normality were very close to those for the 4-chamber TA diameter obtained with the 3DE software package. This suggests that the RV-focused view is more representative than the conventional 4-chamber view by 2DE of the largest TA septal-lateral distance and very similar to the one depicted in the “user-defined” 4-chamber view cut-plane, which is manually aligned, selected, and verified when using 4DAutoTVQ software package on 3DE datasets.

The sphericity index of TA obtained from our healthy subjects was similar to that obtained in other 3DE studies (9, 24, 40), showing an elliptical shape of TA rather than a circular one previously identified in patients with functional TR (29).

In our subjects, absolute TA 3D area, perimeter, diameters, coaptation height, maximum tenting height, and tenting volume were larger in men than women. However, sex-related differences persisted only for the TA area and 4-chamber diameter after indexation for BSA. This finding may have important clinical implications since current guidelines identify a single threshold value of the 4-chamber diameter for both sexes to diagnose TA dilation (5, 6, 8, 23). Moreover, the TA sphericity index and excursion were similar between the sexes. Addetia et al. (9) also reported larger 3D TA area, perimeters, and diameters in men, but after indexing to BSA, both the perimeters and long-axis dimensions became smaller in men than in women. We could not confirm this finding in our healthy subjects. Finally, similar to other studies (9, 24), we found no age-related differences in TA parameters.

### Normal TA dynamics

Similar to other studies (24, 41, 42), we found that 3DE TA parameters increased from mid-systole to early diastole, reaching a maximum in end-diastole. Fukuda et al. (29) reported an interesting dynamic change of 3D TA, with minimum values in mid-systole and a biphasic pattern with two peaks in early and late diastole. However, their cohort included only 15 healthy volunteers.

Conversely, Addetia et al. (9) showed a progressive decrease of TA 3D area, perimeter, and diameters during systole, with a minimum in the end-systolic frame and the largest measurements in late diastole. In addition, in a 2DE study, TA diameters reached the maximum values in early diastole (11).

Using 3DE semi-automated quantitative analysis, we demonstrated that TA enlarged progressively from mid-systole to late diastole, with TA showing a more oval shape during diastole, like other 3DE studies (9, 40). Conversely, a 3DE study that used tomographic cut planes obtained from 3DE datasets to assess TA area and diameters, reported TA sphericity index with no significant variation during the cardiac cycle (24). However, the 2D cut planes cannot account for the complex geometric changes occurring in a saddle-shaped anatomical structure.

### Relationship between TA size and right heart chamber volumes

Anwar et al. (39) identified a strong relationship between TA size assessed by both 3DE and CMR and RV function in healthy subjects. Addetia et al. (9) and Miglioranza et al. (24) demonstrated that 2D and 3D TA dimensions correlated with both RA and RV volumes. Our findings also showed a significant correlation of 3DE TA parameters with maximal RA maximal volume. This is not an unexpected result because it has been proven that an enlarged RA secondary to atrial fibrillation favors TA dilatation and the occurrence of functional TR (2, 3, 43–47). Moreover, we demonstrated that RA maximal volume was independently associated with TA 3D area both at end-diastole and mid-systole.

Finally, the good intra- and interobserver reproducibility and feasibility support the clinical value of the dedicated 3DE software for the assessment of TA geometry parameters.

### Study limitations

Our population consisted of Caucasian healthy volunteers only, which could limit the applicability of our reference value to other ethnic groups. TV data set acquisitions, and quantitative analyses were performed using a single vendor platform, which may limit the generalizability of the results. However, to date, there is no other commercially available software tool dedicated to TV quantification to compare our 3DE reference values.

## Conclusions

In healthy volunteers, reference values for 3DE TA parameters should be gender specific and indexed to BSA. TA dimensions correlate with right chamber volumes. Dynamic changes during the cardiac cycle identify a minimum TA size in mid-systole, then increasing during early-diastole and reaching a maximum value during end-diastole. Conventional 2DE underestimates true 3DE TA diameters. Thus, a comprehensive static and dynamic analysis of TA could improve the understanding of TR pathophysiology and the accuracy of preoperative planning for TV repair.

## Data availability statement

The raw data supporting the conclusions of this article will be made available by the authors, without undue reservation.

## Ethics statement

The studies involving human participants were reviewed and approved by Istituto Auxologico Ethics Committee (Record #2020_04_21_06, approved on April 21, 2020). The patients/participants provided their written informed consent to participate in this study.

## Author contributions

DMu designed the study, interpreted the results, and drafted the manuscript. MG designed the validation study with cardiac CT and revised the manuscript critically for important intellectual content. FH analyzed the cardiac CT data, the 3D datasets of patients included in the validation study, and revised critically the manuscript for important intellectual content. DMi analyzed the 3DE datasets of the subjects included in the normative study, subjects used for assessing the intra-observer variability of our results, and revised critically the manuscript for important intellectual content. AG designed the study, analyzed the 3DE datasets of the subjects used to assess the inter-observer variability of our results, and revised the manuscript critically for important intellectual content. NR designed the database, analyzed the data, and revised the manuscript critically for important intellectual content. GM enrolled patients for the validation study and revised the manuscript critically for important intellectual content. MT, SS, and GP revised the manuscript critically for important intellectual content. LB designed the study, interpreted the results, and revised the manuscript critically for important intellectual content. All authors approved the final manuscript.

## Funding

This study was partially supported by the Italian Ministry of Health.

## Conflict of interest

Author MG received personal fees from Abbot Vascular. Authors DMu and LB are members of the speaker bureaus of GE Healthcare and Philips Medical Systems and received research grants from GE Healthcare, Philips Medical Systems, EsaOte, and TomTec. The remaining authors declare that the research was conducted in the absence of any commercial or financial relationships that could be construed as a potential conflict of interest.

## Publisher's note

All claims expressed in this article are solely those of the authors and do not necessarily represent those of their affiliated organizations, or those of the publisher, the editors and the reviewers. Any product that may be evaluated in this article, or claim that may be made by its manufacturer, is not guaranteed or endorsed by the publisher.

## References

[1] Florescu DR Muraru D Volpato V Gavazzoni M Caravita S Tomaselli M. Atrial functional tricuspid regurgitation as a distinct pathophysiological and clinical entity: no idiopathic tricuspid regurgitation anymore. J Clin Med. (2022) 11:382. 10.3390/jcm11020382 35054074 PMC8781398

[2] Guta AC Badano LP Tomaselli M Mihalcea D Bartos D Parati G. The pathophysiological link between right atrial remodeling and functional tricuspid regurgitation in patients with atrial fibrillation: a three-dimensional echocardiography study. J Am Soc Echocardiogr. (2021) 34:585–94.e1. 10.1016/j.echo.2021.01.004 33440232

[3] Muraru D Caravita S Guta AC Mihalcea D Branzi G Parati G. Functional tricuspid regurgitation and atrial fibrillation: which comes first, the chicken or the egg? Case. (2020) 4:458–63. 10.1016/j.case.2020.04.011 33117949 PMC7581628

[4] Muraru D Guta AC Ochoa-Jimenez RC Bartos D Aruta P Mihaila S. Functional regurgitation of atrioventricular valves and atrial fibrillation: an elusive pathophysiological link deserving further attention. J Am Soc Echocardiogr. (2020) 33:42–53. 10.1016/j.echo.2019.08.016 31685293

[5] Writing Committee M Otto CM Nishimura RA Bonow RO Carabello BA Erwin JP III. 2020 ACC/AHA guideline for the management of patients with valvular heart disease: a report of the American College of Cardiology/American Heart Association Joint Committee on Clinical Practice Guidelines. J Thorac Cardiovasc Surg. (2021) 162:e183–353. 10.1016/j.jtcvs.2021.04.002 33972115

[6] Vahanian A Beyersdorf F Praz F Milojevic M Baldus S Bauersachs J. (2021). ESC/EACTS Guidelines for the management of valvular heart disease. Eur Heart J. (2021) 43:561–632. 10.1093/ejcts/ezac209 34453165

[7] Russo G Taramasso M Pedicino D Gennari M Gavazzoni M Pozzoli A. Challenges and future perspectives of transcatheter tricuspid valve interventions: adopt old strategies or adapt to new opportunities? Eur J Heart Fail. (2022) 24:442–54. 10.1002/ejhf.2398 34894039

[8] Lancellotti P Pibarot P Chambers J La Canna G Pepi M Dulgheru R. Multi-modality imaging assessment of native valvular regurgitation: an EACVI and ESC council of valvular heart disease position paper. Eur Heart J Cardiovasc Imaging. (2022) 23:e171–232. 10.1093/ehjci/jeab253 35292799

[9] Addetia K Muraru D Veronesi F Jenei C Cavalli G Besser SA. 3-dimensional echocardiographic analysis of the tricuspid annulus provides new insights into tricuspid valve geometry and dynamics. JACC Cardiovasc Imag. (2019) 12:401–12. 10.1016/j.jcmg.2017.08.022 29153573

[10] Knio ZO Montealegre-Gallegos M Yeh L Chaudary B Jeganathan J Matyal R. Tricuspid annulus: a spatial and temporal analysis. Ann Card Anaesth. (2016) 19:599–605. 10.4103/0971-9784.191569 27716689 PMC5070318

[11] Miglioranza MH Mihaila S Muraru D Cucchini U Iliceto S Badano LP. Variability of tricuspid annulus diameter measurement in healthy volunteers. JACC Cardiovasc Imag. (2015) 8:864–6. 10.1016/j.jcmg.2014.09.010 25459303

[12] Muraru D Hahn RT Soliman OI Faletra FF Basso C Badano LP. 3-Dimensional echocardiography in imaging the tricuspid valve. JACC Cardiovasc Imag. (2019) 12:500–15. 10.1016/j.jcmg.2018.10.035 30846124

[13] Badano LP Hahn R Rodriguez-Zanella H Araiza Garaygordobil D Ochoa-Jimenez RC Muraru D. Morphological assessment of the tricuspid apparatus and grading regurgitation severity in patients with functional tricuspid regurgitation: thinking outside the box. JACC Cardiovasc Imag. (2019) 12:652–64. 10.1016/j.jcmg.2018.09.029 30947907

[14] Peluso D Badano LP Muraru D Dal Bianco L Cucchini U Kocabay G. Right atrial size and function assessed with three-dimensional and speckle-tracking echocardiography in 200 healthy volunteers. Eur Heart J Cardiovasc Imaging. (2013) 14:1106–14. 10.1093/ehjci/jet024 23423966

[15] Maffessanti F Muraru D Esposito R Gripari P Ermacora D Santoro C. Age-, body size-, and sex-specific reference values for right ventricular volumes and ejection fraction by three-dimensional echocardiography: a multicenter echocardiographic study in 507 healthy volunteers. Circ Cardiovasc Imag. (2013) 6:700–10. 10.1161/CIRCIMAGING.113.000706 23811752

[16] Muraru D Maffessanti F Kocabay G Peluso D Dal Bianco L Piasentini E. Ascending aorta diameters measured by echocardiography using both leading edge-to-leading edge and inner edge-to-inner edge conventions in healthy volunteers. Eur Heart J Cardiovasc Imaging. (2014) 15:415–22. 10.1093/ehjci/jet173 24096712

[17] Lang RM Badano LP Tsang W Adams DH Agricola E Buck T. EAE/ASE recommendations for image acquisition and display using three-dimensional echocardiography. Eur Heart J Cardiovasc Imaging. (2012) 13:1–46. 10.1093/ehjci/jer316 22275509

[18] Muraru D Spadotto V Cecchetto A Romeo G Aruta P Ermacora D. New speckle-tracking algorithm for right ventricular volume analysis from three-dimensional echocardiographic data sets: validation with cardiac magnetic resonance and comparison with the previous analysis tool. Eur Heart J Cardiovasc Imag. (2016) 17:1279–89. 10.1093/ehjci/jev309 26647080

[19] Florescu DR Badano LP Tomaselli M Torlasco C Tartea GC Balseanu TA. Automated left atrial volume measurement by two-dimensional speckle-tracking echocardiography: feasibility, accuracy, and reproducibility. Eur Heart J Cardiovasc Imag. (2021) 23:85–94. 10.1093/ehjci/jeab199 34606605

[20] Ahn Y Koo HJ Kang JW Yang DH. Tricuspid valve imaging and right ventricular function analysis using cardiac CT and MRI. Korean J Radiol. (2021) 22:1946–63. 10.3348/kjr.2020.1507 34668349 PMC8628151

[21] Pontone G Muscogiuri G Andreini D Guaricci AI Guglielmo M Baggiano A. Impact of a new adaptive statistical iterative reconstruction (ASIR)-V algorithm on image quality in coronary computed tomography angiography. Acad Radiol. (2018) 25:1305–13. 10.1016/j.acra.2018.02.009 29602723

[22] van Rosendael PJ Kamperidis V Kong WK van Rosendael AR van der Kley F Ajmone Marsan N. Computed tomography for planning transcatheter tricuspid valve therapy. Eur Heart J. (2016) 38:665–74. 10.1093/eurheartj/ehw499 27807057

[23] Hahn RT Badano LP Bartko PE Muraru D Maisano F Zamorano JL. Tricuspid regurgitation: recent advances in understanding pathophysiology, severity grading and outcome. Eur Heart J Cardiovasc Imag. (2022) 23:913–29. 10.1093/ehjci/jeac009 35157070

[24] Miglioranza MH Mihaila S Muraru D Cucchini U Iliceto S Badano LP. Dynamic changes in tricuspid annular diameter measurement in relation to the echocardiographic view and timing during the cardiac cycle. J Am Soc Echocardiogr. (2015) 28:226–35. 10.1016/j.echo.2014.09.017 25450013

[25] Foale R Nihoyannopoulos P McKenna W Kleinebenne A Nadazdin A Rowland E. Echocardiographic measurement of the normal adult right ventricle. Br Heart J. (1986) 56:33–44. 10.1136/hrt.56.1.33 3730205 PMC1277383

[26] Zhan Y Debs D Khan MA Nguyen DT Graviss EA Shah DJ. Normal reference values and reproducibility of tricuspid annulus dimensions using cardiovascular magnetic resonance. Am J Cardiol. (2019) 124:594–8. 10.1016/j.amjcard.2019.05.019 31208699

[27] Ricci F Aung N Gallina S Zemrak F Fung K Bisaccia G. Cardiovascular magnetic resonance reference values of mitral and tricuspid annular dimensions: the UK Biobank cohort. J Cardiovasc Magn Reson. (2020) 23:5. 10.1186/s12968-020-00688-y 33407573 PMC7788733

[28] Ton-Nu TT Levine RA Handschumacher MD Dorer DJ Yosefy C Fan D. Geometric determinants of functional tricuspid regurgitation:insights from 3-dimensional echocardiography. Circulation. (2006) 114:143–9. 10.1161/CIRCULATIONAHA.106.611889 16818811

[29] Fukuda S Saracino G Matsumura Y Daimon M Tran H Greenberg NL. Three-dimensional geometry of the tricuspid annulus in healthy subjects and in patients with functional tricuspid regurgitation: a real-time, 3-dimensional echocardiographic study. Circulation. (2006) 114:I492–8. 10.1161/CIRCULATIONAHA.105.000257 16820625

[30] Genovese D Mor-Avi V Palermo C Muraru D Volpato V Kruse E. Comparison between four-chamber and right ventricular-focused views for the quantitative evaluation of right ventricular size and function. J Am Soc Echocardiogr. (2019) 32:484–94. 10.1016/j.echo.2018.11.014 30686498

[31] Zamorano JL Badano LP Bruce C Chan KL Goncalves A Hahn RT. EAE/ASE recommendations for the use of echocardiography in new transcatheter interventions for valvular heart disease. Eur J Echocardiogr. (2011) 12:557–84. 10.1093/ejechocard/jer086 21841044

[32] Dreyfus J Durand-Viel G Raffoul R Alkhoder S Hvass U Radu C. Comparison of 2-dimensional, 3-dimensional, and surgical measurements of the tricuspid annulus size: clinical implications. Circ Cardiovasc Imag. (2015) 8:e003241. 10.1161/CIRCIMAGING.114.003241 26156015

[33] Agoston G Gargani L Miglioranza MH Caputo M Badano LP Moreo A. Left atrial dysfunction detected by speckle tracking in patients with systemic sclerosis. Cardiovasc Ultrasound. (2014) 12:30. 10.1186/1476-7120-12-30 25090937 PMC4134332

[34] Caravita S Figliozzi S Florescu DR Volpato V Oliverio G Tomaselli M. Recent advances in multimodality imaging of the tricuspid valve. Expert Rev Med Devices. (2021) 18:1069–81. 10.1080/17434440.2021.1990753 34617481

[35] Badano LP Caravita S Rella V Guida V Parati G Muraru D. The added value of 3-dimensional echocardiography to understand the pathophysiology of functional tricuspid regurgitation. JACC Cardiovasc Imag. (2021) 14:683–9. 10.1016/j.jcmg.2020.04.029 32682722

[36] Khalique OK Cavalcante JL Shah D Guta AC Zhan Y Piazza N. Multimodality imaging of the tricuspid valve and right heart anatomy. JACC Cardiovasc Imag. (2019) 12:516–31. 10.1016/j.jcmg.2019.01.006 30846125

[37] Muraru D Veronesi F Maddalozzo A Dequal D Frajhof L Rabischoffsky A. 3D printing of normal and pathologic tricuspid valves from transthoracic 3D echocardiography data sets. Eur Hear J Cardiovasc Imag. (2017) 18:802–8. 10.1093/ehjci/jew215 28025262

[38] Muraru D. 22nd annual feigenbaum lecture: right heart, right now: the role of three-dimensional echocardiography. J Am Soc Echocardiogr. (2022). (in press). 10.1016/j.echo.2022.05.011 35644303

[39] Anwar AM Geleijnse ML Soliman OI McGhie JS Frowijn R Nemes A. Assessment of normal tricuspid valve anatomy in adults by real-time three-dimensional echocardiography. Int J Cardiovasc Imaging. (2007) 23:717–24. 10.1007/s10554-007-9210-3 17318363 PMC2048827

[40] Utsunomiya H Itabashi Y Kobayashi S Rader F Siegel RJ Shiota T. Clinical impact of size, shape, and orientation of the tricuspid annulus in tricuspid regurgitation as assessed by three-dimensional echocardiography. J Am Soc Echocardiogr. (2020) 33:191–200.e1. 10.1016/j.echo.2019.09.016 31837928

[41] Anwar AM Soliman OI Nemes A van Geuns RJ Geleijnse ML Ten Cate FJ. Value of assessment of tricuspid annulus: real-time three-dimensional echocardiography and magnetic resonance imaging. Int J Cardiovasc Imag. (2007) 23:701–5. 10.1007/s10554-006-9206-4 17295104 PMC2048828

[42] Owais K Taylor CE Jiang L Khabbaz KR Montealegre-Gallegos M Matyal R. Tricuspid annulus: a three-dimensional deconstruction and reconstruction. Ann Thorac Surg. (2014) 98:1536–42. 10.1016/j.athoracsur.2014.07.005 25249160 PMC6563329

[43] Ortiz-Leon XA Posada-Martinez EL Trejo-Paredes MC Ivey-Miranda JB Pereira J Crandall I. Understanding tricuspid valve remodelling in atrial fibrillation using three-dimensional echocardiography. Eur Hear J Cardiovasc Imaging. (2020) 21:747–55. 10.1093/ehjci/jeaa058 32372089

[44] Utsunomiya H Itabashi Y Mihara H Berdejo J Kobayashi S Siegel RJ. Functional tricuspid regurgitation caused by chronic atrial fibrillation: a real-time 3-dimensional transesophageal echocardiography study. Circ Cardiovasc Imag. (2017) 10:e004897. 10.1161/CIRCIMAGING.116.004897 28073806

[45] Florescu DR Muraru D Florescu C Volpato V Caravita S Perger E. Right heart chambers geometry and function in patients with the atrial and the ventricular phenotypes of functional tricuspid regurgitation. Eur Hear J Cardiovasc Imag. (2022) 23:930–40. 10.1093/ehjci/jeab211 34747460

[46] Muraru D Addetia K Guta AC Ochoa-Jimenez RC Genovese D Veronesi F. Right atrial volume is a major determinant of tricuspid annulus area in functional tricuspid regurgitation: a three-dimensional echocardiographic study. Eur Hear J Cardiovasc Imag. (2021) 22:660–9. 10.1093/ehjci/jeaa286 33387441

[47] Muraru D Parati G Badano LP. Does atrial fibrillation affect the tricuspid annulus 3D geometry in patients without severe valve regurgitation? Eur Hear J Cardiovasc Imag. (2020) 21:756–8. 10.1093/ehjci/jeaa082 32402062

